# Highly Selective Polypyrrole MIP-Based Gravimetric and Electrochemical Sensors for Picomolar Detection of Glyphosate

**DOI:** 10.3390/s17112586

**Published:** 2017-11-09

**Authors:** Zouhour Mazouz, Seyfeddine Rahali, Najla Fourati, Chouki Zerrouki, Nadia Aloui, Mahamadou Seydou, Nourdin Yaakoubi, Mohamed M. Chehimi, Ali Othmane, Rafik Kalfat

**Affiliations:** 1 Institut National de Recherches et d’Analyses Physico-Chimiques, Laboratoire Matériaux, Traitement et Analyse, BiotechPole Sidi-Thabet, 2032 Ariana, Tunisia; mazouz.zouhour645@gmail.com (Z.M.); rafik.kalfat@gmail.com (R.K.); 2 Unité de Recherche en Modélisation des Sciences Fondamentales et Didactiques, Université de Tunis El Manar, Tunis, Campus Universitaire Farhat-Hached Tunis, 1068 Rommana, Tunisia; saif.rahali@gmail.com; 3 Cnam, SATIE, UMR CNRS 8029, 292 Rue Saint Martin, 75003 Paris, France; zerrouki@cnam.fr; 4 LAUM, UMR CNRS 6613, Université du Maine, Avenue Olivier Messiaen, 72085 Le Mans CEDEX 9, France; nadia.aloui@univ-lemans.fr (N.A.); nourdin.yaakoubi@univ-lemans.fr (N.Y.); 5 ITODYS, UMR CNRS 7086, Université Paris Sorbonne Paris Cité, 15 Rue J-A de Baïf, 75013 Paris, France; mahamadou.seydou@univ-paris-diderot.fr; 6 CNRS, ICMPE, UMR CNRS 7182, 2-8 rue Henri Dunant, 94320 Thiais, France; chehimi@icmpe.cnrs.fr; 7 Faculté de Médecine de Monastir, Laboratoire d’Interfaçe et de Matériaux Avancés, Université de Monastir, Av. Avicenne, 5000 Monastir, Tunisia; ali.othmane54@gmail.com

**Keywords:** glyphosate, molecularly imprinted polymer (MIP), gravimetric sensor, electrochemical sensor, DFT calculation, dissociation constants

## Abstract

There is a global debate and concern about the use of glyphosate (Gly) as an herbicide. New toxicological studies will determine its use in the future under new strict conditions or its replacement by alternative synthetic or natural herbicides. In this context, we designed biomimetic polymer sensing layers for the selective molecular recognition of Gly. Towards this end, complementary surface acoustic wave (SAW) and electrochemical sensors were functionalized with polypyrrole (PPy)-imprinted polymer for the selective detection of Gly. Their corresponding limits of detection were on the order of 1 pM, which are among the lowest values ever reported in literature. The relevant dissociation constants between PPy and Gly were estimated at [K_d1_ = (0.7 ± 0.3) pM and K_d2_ = (1.6 ± 1.4) µM] and [K_d1_ = (2.4 ± 0.9) pM and K_d2_ = (0.3 ± 0.1) µM] for electrochemical and gravimetric measurements, respectively. Quantum chemical calculations permitted to estimate the interaction energy between Gly and PPy film: ΔE = −145 kJ/mol. Selectivity and competitivity tests were investigated with the most common pesticides. This work conclusively shows that gravimetric and electrochemical results indicate that both MIP-based sensors are perfectly able to detect and distinguish glyphosate without any ambiguity.

## 1. Introduction

Glyphosate (Gly) was introduced to the consumer market in 1974 as a broad-spectrum herbicide and quickly became one of the best-selling herbicides [1]. The introduction of genetically-engineered glyphosate-tolerant crops in the last twenty years has increased its use considerably in agriculture and non-agricultural applications [2]. Glyphosate is chemically stable in water and is not subject to photochemical degradation. It can diffuse into surface and sub-surface waters by direct use near aquatic environments or by runoff or leaching from terrestrial applications [3,4]. Once in the environment, glyphosate is adsorbed to soil and is broken down by soil microbes to its major metabolite amino methyl phosphonic acid (AMPA) [4]. Humans, animals, plants, and foods may, thus, be easily contaminated, causing consequently a great environmental impact in all aspects. The hazard potential of glyphosate is still unclear, as it possesses amino acid-like structures which might interfere with the formation of amino acids and other chemicals in plants. In 2015, the WHO International Agency for Research on Cancer classified glyphosate as “probably carcinogenic to humans” [5]. Several other studies have demonstrated the potential of glyphosate to be an endocrine disruptor [2,6,7]. Thus, international organizations have tried to regulate the levels of Gly admitted in the environment. The European Union proposed a required limit in drinking water equal to 0.1 μg L^−1^ (5.9 × 10^−10^ M). These health concerns have motivated the development of numerous analytical methods to track glyphosate in water samples, dust, air, soil, plant vegetation, and body fluids. Examples of investigated analytical methods are presented in Table 1.

Despite their high sensitive and accurate detection, many of the developed methods need labels, are time-consuming, and/or require extensive instrumentation. An interesting alternative approach consists of the design of sensors functionalized with molecularly-imprinted polymer (MIP) for the selective detection of glyphosate. The rationale of this strategy is related to the fact that molecular imprinting is low-cost, sensitive, selective, specific, and versatile [21,22,23,24].

Molecular imprinting is based on the selective recognition of a specific target because of the dedicated architecture of cavities embedded in the polymer matrix. It generally involves the following steps: (i) a prearrangement of the functional monomers and crosslinking molecules around the template; (ii) polymerization of the resulting complex; and (iii) template removal from the vicinity of the synthesized MIP by solvent extraction or chemical cleavage. The created cavities act as highly-specific receptors which are complementary to the templates in terms of size, shape, and arrangement of functional groups [25,26,27,28].

Here, the MIP consists of a polypyrrol matrix (PPy), in which Gly templates were embedded. PPy was chosen among the large variety of conducting polymers for several reasons: its stability in a wide range of pH, the stability over time of the synthetized films, and the ease of electropolymerization on various substrates. Muzyka et al. reported that the stability of the created imprinted sites within such polymer is related to the irreversible PPy overoxidation and that the recognition properties are attributable to the incorporation of carbonyl and carboxylic groups the polypyrrole backbone during the overoxidation process. According to the authors, the PPy-template hydrogen bonding, e.g., between N–H group of pyrrole and some functional groups, such as C=O group or nitrogen atoms in the template, account for the selectivity of the sensor towards the said analyte. However, such hypotheses can best be verified via computational chemistry methods, such as density functional theory (DFT), in order to assess the conformational and chemical complementarity between Gly templates and two pesticides interferents (simetryn and omethoate) with the PPy matrix. Compared to other studies, here we have described the PPy polymer as two layers separated by a certain distance, and not by a limited number of pyrrole monomers [29,30,31].

Two transduction techniques were investigated in this study: gravimetry and electrochemistry. Several reasons have motivated this choice: (i) primarily, these two methods are label free, sensitive, reliable, and allow reaching low limits of detection (LOD); (ii) secondly, redundancy is an efficient means that can eliminate all experimental biases and permits validation of the obtained results; (iii) finally, these techniques allow obtaining complementary information: electrochemistry permits the characterization of the investigated surfaces after each functionalization step, a key element to ensure the correct functioning of a given sensor, while gravimetry allows determining and quantifying the recognition kinetics between the matrix and an investigated analyte [32,33]. 

To the best of our knowledge, this is the first study concerning the detection of Gly with a surface acoustic wave sensor functionalized with a molecular imprinted polymer, and the first work that permits the estimation of dissociation constant values between the polypyrrole matrix and Gly from two different transductions techniques.

Selectivity and competitivity were investigated with gluphosinate ammonium, simetryn, terbuthylazine-2-hydroxy, omethoate, and methidathion, pesticides which usually coexist with Gly.

## 2. Materials and Methods

### 2.1. Chemicals

Sulfuric acid (H_2_SO_4_, 95%), acetic acid (CH_3_COOH), hydrogen peroxide (H_2_O_2_, 30%), methanol (CH_3_OH), lithium perchlorate (LiClO_4_) glyphosate (C_3_H_8_NO_5_P), gluphosinate ammonium (C_3_H_11_N_2_O_5_P), simetryn (C_8_H_15_N_5_S), terbuthylazine-2-hydroxy (C_9_H_17_N_5_O), omethoate (C_5_H_12_NO_4_PS) and methidathion (C_6_H_11_N_2_O_4_PS_3_) were purchased from Sigma Aldrich (Lyon, France) and used as received. Pyrrole (Py) was purified before its use by filtering through basic alumina column and stored in dark at 4 °C. Lithium perchlorate was used as supporting electrolyte in all electrochemical measurements.

### 2.2. Instrumentation

#### 2.2.1. Gravimetric Measurements

Shear horizontal surface acoustic wave (SH-SAW) devices (104 MHz) were fabricated on 36° rotated lithium tantalate (LiTaO_3_) piezoelectric substrates. Both interdigital transducers (IDTs) and the sensitive area (zone separating emitters and receivers IDTs) were realized by evaporating 20/80 nm Cr/Au thin layers on LiTaO_3_ surfaces. A HP8214 network analyzer was used to follow up the temporal variations of the phase and modulus of the output signal, after template extraction and further analyte injection. 

#### 2.2.2. Electrochemical Measurements

Chronamperometry (CA) and square wave voltammetry (SWV) measurements were performed with a CHI 650E electrochemical workstation (CH Instrument Inc., IJ Cambria Scientific Ltd., Llwynhendy, UK) and a three-electrode configuration: a gold electrode, a saturated calomel electrode (SCE), and a platinum wire as the working, the reference and the auxiliary electrodes, respectively. Depending on the investigated technique, the working electrode (S = 22 mm^2^) was either the SAW sensor sensing area or a gold electrode. All experiments were carried out at room temperature and under ambient air.

In this study, chronoamperometry was used to electropolymerize the MIPs on the sensing area of both electrochemical and gravimetric sensors, while square wave voltammetry (SWV) was investigated to optimize MIP synthesizing parameters and to follow up Gly template extraction and analyte detection (Gly and other pesticides molecules). For all the SWV measurements, we have used the following parameters: increment = 5 mV, amplitude = 10 mV, frequency = 25 Hz, quiet time = 5 s, and sensitivity = 1 × 10^−4^.

#### 2.2.3. AFM Measurements

AFM measurements were carried out under ambient conditions with a Nanosurf easyScan 2 Flex system, in the phase contrast mode, equipped with commercially Tap190Al-G probes (from Budget Sensors, Sofia, Bulgaria). The cantilever’s resonance frequency was of about 190 kHz and the AFM probe’s curvature radius was less than 10 nm.

#### 2.2.4. Calculation Methods

Calculations were performed using the method of density functional theory (DFT) in periodic conditions provided by means of code “VASP 5.2.11” (Vienna Ab Initio Simulation Package) [34,35,36]. The electron-ion interactions have been described by the “PAW” method (projector augmented wave) [37,38]. The convergence of the expansion of the plane wave is obtained with a cut-off of 500 eV. The Generalized gradient approximation (GGA) was used with functional Perdew-Burke-Ernzerhof (PBE) [39]. The sampling in the Brillouin zone has been carried out on a grid of 3 × 3 × 3 k-points. All of the computations reported in this paper are performed using the dispersion-including DFT Grimme D3 method [40].

## 3. Results

### 3.1. MIP Design

Prior to any measurement, a drop of a piranha solution (98% H_2_SO_4_/30% H_2_O_2_ 1:1 *v*/*v*) was deposited, during 20 min, on the gold sensing areas of both SAW and electrochemical transducers. The substrates were then copiously rinsed with deionized double distilled (DI) water, then with ethanol before being dried under ambient air.

As demonstrated in our previous studies [21,22], electrodepositing a thin polypyrrole blocking layer (BL) on a gold electrode is necessary to prevent the formation of complexes with gold. This primer layer has also the advantage to lower the oxidation potential of pyrrole during the preparation of the MIP [22]. Here, we have prepared the BL by CA at a constant potential of 1.05 V vs. SCE during 2 s. 

MIPs were then prepared by CA at a fixed potential of 1.05 V vs. SCE during 5 s with the following concentrations [PPy] = 10^−2^ M, [Gly] = 10^−4^ M and [LiClO_4_] = 10^−2^ M. The pH of the prepolymerization solution was equal to 5.

**Figure 1:**
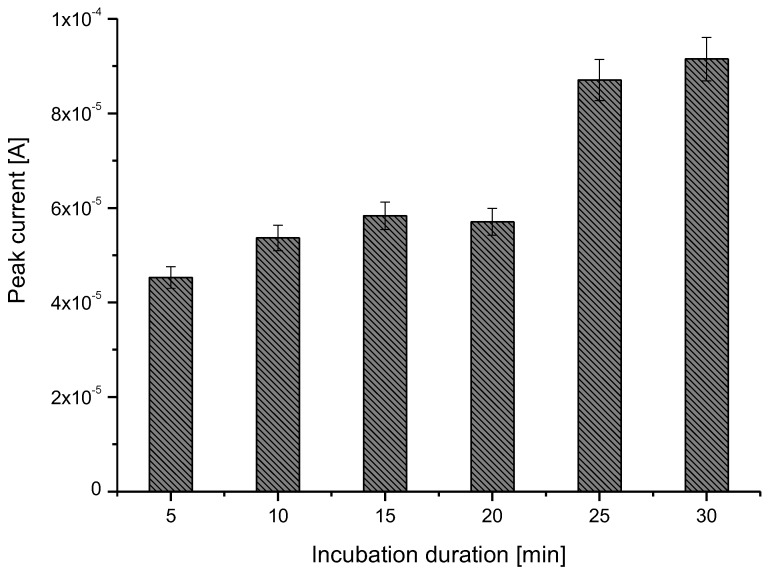
Monitoring of glyphosate oxidation peak current variation versus incubation duration in a pyrrole/glyphosate/LiClO_4_ solution.

Several SWV measurements were performed to determine the optimal incubation duration of the templates in a pyrrole solution. Results, presented in Figure 1, show that the Gly oxidation peak increases with time and levels off at about 25 min. We have, thus, chosen an incubation duration of 30 min for MIP realization and further Gly detection. 

Thin films of non-imprinted polypyrrole polymer (NIP) have also been prepared under identical electropolymerization conditions but without Gly, in order to evaluate nonspecific adsorptions.

### 3.2. Glyphosate Extraction

**Figure 2:**
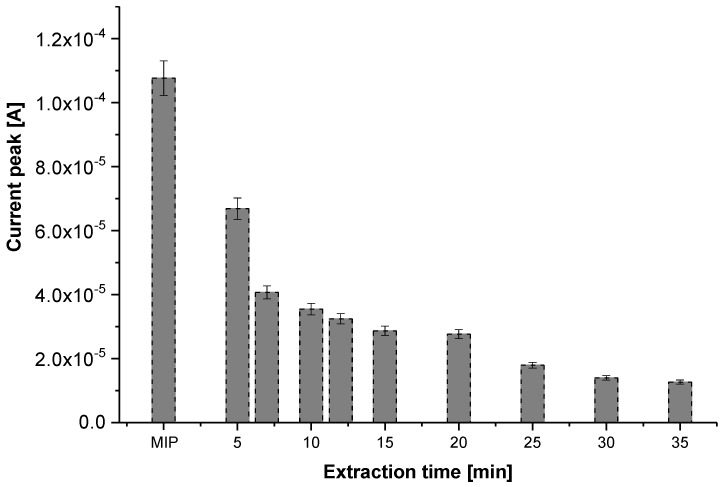
Follow-up of the peak current variation versus the extraction duration in methanol.

**Figure 3:**
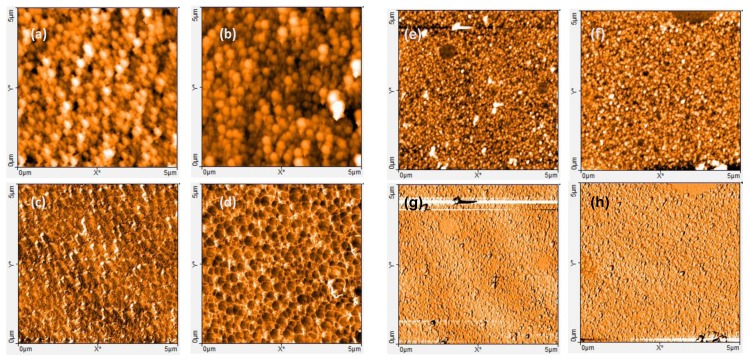
AFM images (5 × 5 µm) of MIP (**a**–**d**) and NIP (**e**–**h**), before (**a**,**c**,**e**,**g**) and after (**b**,**d**,**f**,**h**) immersion in the protic solution. Topographic mode (top) and phase contrast mode (bottom).

The gold coated electrodes, intended to electrochemical measurements, were entirely dipped in the protic solution (methanol/acetic acid 1:1 *v*/*v*), then in deionized double distilled (DI) water during 30 min, before being dried in air. The follow up of variations of peak currents (determined from SWV measurements), versus extraction duration in the protic solution, indicate that extracting Gly during 30 min is the most appropriate compromise between template extraction and keeping intact the PPy matrix. Actually, a duration of 30 min permits the reduction in the current peak attributed to Gly oxidation by about 88% (Figure 2) without altering the morphological structure of the MIPs, as shown in the AFM images of Figure 3.

AFM images displayed in Figure 3 present the morphological structures of the MIP, NIP before (a, c, e, g) and after (b, d, f, h) immersion in the protic solution. The extraction of Gly templates from the vicinity of the MIP causes an important structural change as revealed by the alveolar-like character in the phase contrast image (Figure 3d). To ensure that the protic solution extracts only Gly templates and does not modify the film itself, we have immersed the NIP in the methanol/acetic acid solution during 30 min. AFM images realized before and after immersion in this solution are presented in Figure 3. As expected, it appears that contrary to MIP, the protic solution does not affect the NIP structure. This highlights the efficiency of the involved procedure to extract only Gly templates without altering the PPy matrix.

For gravimetric measurements, a continuous flow of the protic solution was injected over the SAW sensor sensing area at a constant flow rate of 0.19 mL min^−1^. The extractor solvent was then drained and replaced by a continuous flow of DI water for 30 min to rinse the sensing area. The follow up of phase variation versus time showed an increase of phase values indicating that glyphoaste molecules were removed from the PPy matrix (results not shown here).

### 3.3. Glyphosate Detection

Prior to Gly detection, we have tried to assess the possible effects of non-specific adsorption between Gly analytes and the polypyrrole matrix. For this purpose, we have prepared two electrodes. The first one was functionalized with a PPy MIP before being extracted with the protic solvent and then incubated during 30 min in a 10^−4^ M solution of Gly. This electrode was characterized by SWV at each of the above-mentioned steps. The second one, coated with NIP, was also characterized by the same technique before and after incubation. 

**Figure 4:**
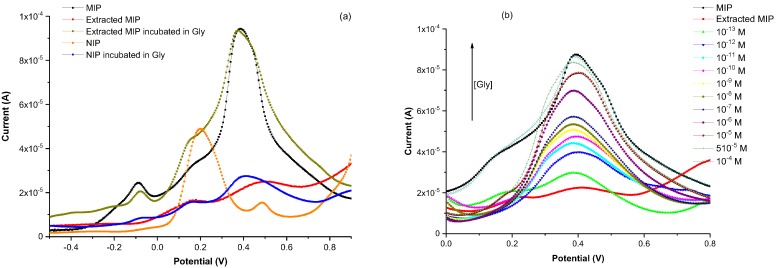
Square wave voltammograms of (**a**) a PPy MIP, before and after Gly extraction, a NIP, and the further extracted MIP incubated in a 10^−4^ M solution of Gly; and (**b**) a MIP-based sensor for various Gly concentrations.

The examination of the voltammograms presented in Figure 4a clearly shows the oxidation peak of Gly at 0.38 V, appearing for the MIP containing the template. This peak is quasi-absent in the response of the extracted MIP (red line), indicating an almost total extraction of the template. The oxidation peak reappears after the incubation of the extracted polymer in a Gly solution, with the same current peak magnitude than the MIP (before extraction). These results indicate that it is possible to perfectly remove and uptake Gly molecules from the vicinity of the designed MIP, thus offering accessible sensing cavities. 

As expected, the voltammogram of the non-imprinted polymer (NIP) incubated in a Gly solution shows a slight oxidation peak, which is comparable to that of the extracted MIP. This can be attributed to the nonspecific adsorption, and can be explained by the fact that at pH 5 (the pH at which the measurements were done), the functional carboxyl groups of Glys are negatively charged and can interact with the positively-charged N-H groups of the pyrrole units present at the surface. This value remains, however, largely lower than that of the MIP after incubation in Gly solution, indicating the weak character of the above-mentioned interaction. In fact, the oxidation peak appearing at about 0.2 V (in the NIP voltammogram) is that of polypyrrole.

In order to evaluate the sensing properties of the designed electrochemical sensor, the extracted MIP-based electrodes were incubated during 30 min in a Gly solution at a considered concentration, then rinsed copiously with DI water. Electrochemical MIP based-sensor’s responses show that the current peak values increase with Gly concentration from 10^−13^ M to 10^−5^ M, and reach saturation at 5 × 10^−5^ M (Figure 4b). The MIP’s functionalized cavities have, thus, recognized the further Gly analytes.

On the basis of our precedent conclusion, concerning the presence of a non-specific adsorption between Gly and the PPy matrix, and on the results presented in Figure 4b, we have estimated the limit of detection of the considered sensor at 10^−12^ M. This LOD is two orders of magnitude lower than the limit value required by the EU for drinking water (0.1 µg/L for pesticides without distinction [41], i.e., 5.9 × 10^−10^ M by transposing to Gly), thus making Gly trace detection, with this designed sensor, possible in the near future. To the best of our knowledge, this LOD is among the lowest values ever reported in the literature (Table 2). To this outstanding performance, we would like to stress the advantage of the simple and efficient protocol described so far for the design of the MIP-based sensor as it needs only one electropolymerization step.

**Figure 5:**
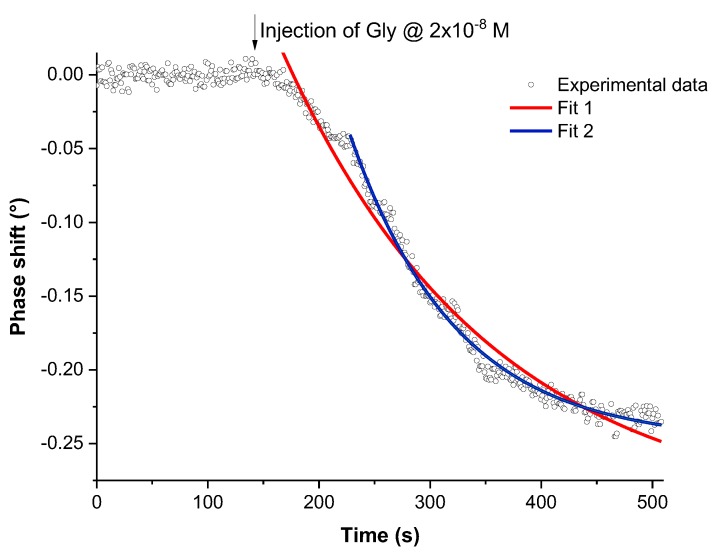
Phase shift versus time variations of the MIP-SAW sensor after the injection of a Gly solution 2 × 10^−8^ M. Experimental results in black dots; exponential decay fit considering the entire recognition time: fit1 in red solid line; and exponential decay fit considering a restricted time domain: fit 2 in blue solid line.

Gly detection has also been investigated by gravimetric technique for concentrations ranging from 10^−12^ M to 4 × 10^−6^ M. All experiments were conducted in DI water without any added salts. The follow up of the time-dependent decrease of the output signal phase, after the injection of Gly solutions, gives access to information on the recognition kinetics of this pesticide by the MIP-SAW sensor (Figure 5). 

The typical shape of the recognition time-dependence can be fitted by an exponential decay. Considering the entire recognition time , the characteristic time constant *τ_c_* is: *τ_c_* = (188 ± 7) s. Excluding the first 40 s, which probably corresponds to the nanoscale rearrangements of the MIP cavities, for a perfect tailoring to the Gly molecules, the recognition process becomes faster: *τ_c_* = (96 ± 2) s. This change in slope suggests the idea of a combined contribution of the structural rearrangement and the chemical complementarities in the recognition processes.

### 3.4. Estimation of Glyphosate/Polypyrrole Dissociation Constants

**Figure 6:**
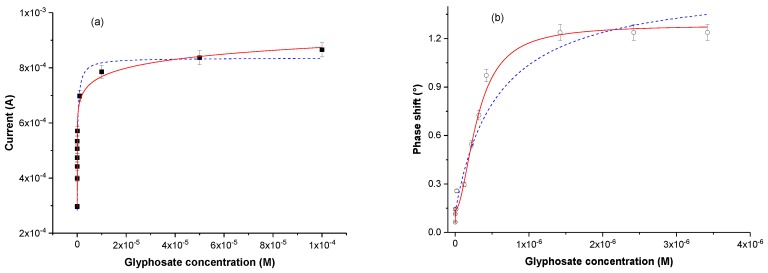
(**a**) Peak current variation versus Gly concentration; comparison between two sites binding model (blue line) and combined model (red line). (**b**) Cumulative absolute values of phase shifts according to Gly concentration (dashed blue line: two sites binding model; solid red line: combined model).

Variations of output signals of electrochemical and gravimetric MIP-based sensors versus Gly concentrations, are presented in Figure 6. Sensitivities of both designed sensors were estimated from successive first injections, i.e., at lower concentrations. They were found on the order of (75 ± 41) µA/nM and (6.9 ± 2.9) × 10^−2^ °/nM for electrochemical and gravimetric sensors, respectively. These sufficiently high values, associated with a low limit of detection (10^−12^ M), allow the consideration of any application for environment monitoring.

Dissociation constants are crucial parameters which permit the estimation of the degree of affinity between the considered polypyrrole MIP and the Gly molecules. Here, gravimetric and electrochemical experimental data were fitted (Figure 6) by a two binding sites model (Equation (1)):

(1) $$y(C)\;=\;\frac{A_{1}\;\times \;C}{K_{d1}\;+\;C}\;+\;\frac{A_{2}\;\times \;C}{K_{d2}\;+\;C}$$

where y(C) corresponds to the output sensor’s signal response (phase or current for gravimetric or electrochemical transduction respectively), for a given Gly concentration C, K_d1_ and K_d2_ are the first and second dissociation constants, A_1_ and A_2_ are empiric constants.

Dissociation constants were estimated at: [K_d1_ = (1.4 ± 0.8) × 10^−12^ M and K_d2_ = (6.7 ± 2.7) × 10^−7^ M] and [K_d1_ = (2.0 ± 0.8) × 10^−12^ M and K_d2_ = (5.7 ± 1.8) × 10^−7^ M] for electrochemical and gravimetric measurements, respectively. The very low value of Kd_1_ confirms the hypothesis of strong bonds between MIP cavities and Gly molecules. However, such a very low value is observed for complementary biological entities than for mimetic systems. In the present case, the recognition phenomena involve not only the complementary functional groups in both matrix and template, but also the size and the shape of the embedded cavities. This implies that recognition can lead to strong bindings where all negative Gly charges bind to pyrrole positives ones. Moreover, the necessary rearrangement of the polypyrrole film around the Gly molecules contribute to the strengthening of these bonds. 

To take into account the polymer mechanical restructuration, at the nanometer scale, we replaced the two sites binding model with a similar one where an exponent is associated with both concentration and dissociation constants (Equation (2)). According to the Hill model, this “ponderation” allows the consideration of an apparent dissociation constant, without drastically changing the initial point of view.

(2) $$y(C)\;=\;\frac{A_{1}\;\times \;C}{K_{d1}\;+\;C}\;+\;\frac{A_{2}\;\times \;C^{\alpha }}{K_{d2}^{\alpha }\;+\;C^{\alpha }}$$

where y(C) is the output sensor’s signal response (phase or current for gravimetric or electrochemical transduction respectively), for a given Gly concentration. C, K_d1_, and K_d2_ are the first and second dissociation constants, A_1_ and A_2_ are empiric constants, and *α* is an empiric ponderation exponent.

According to this novel combined model (Figure 6), the apparent dissociation constants calculated from electrochemical measurements were found to be equal to K_d1_ = (7.1 ± 3.2) × 10^−13^ M and K_d2_ = (1.6 ± 1.4) × 10^−6^ M, whereas those obtained from gravimetric measurements were on the order of K_d1_ = (2.4 ± 0.9) × 10^−12^ M and K_d2_ = (3.0 ± 0.7) × 10^−7^ M. As expected, the novel model fits both electrochemical and gravimetric experimental data better. The estimated K_d_ values are, however, slightly different from those obtained from the two sites binding model.

The slight difference between the dissociation constants estimated from gravimetric and electrochemical measurements can be attributed to the fact that the ionic strength is not the same in the investigated media. In fact, electrochemical measurements are done in solutions containing a relatively high concentration of support electrolyte (LiClO_4_ 10^−1^ M in our case), whereas gravimetric detection is done in DI water.

**Figure 7:**
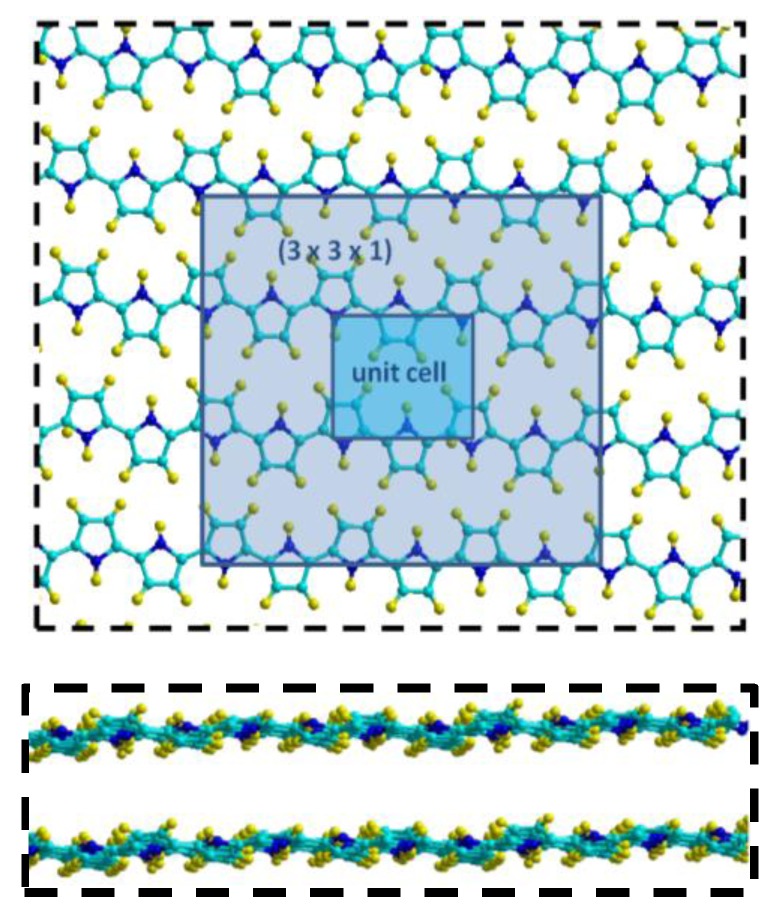
Model of polymer matrix considered for the calculations. The projection in plane shows the primitive lattice and the used super lattice (3 × 3 × 1) (**top**) and the side view show the inter-plane distance (**bottom**).

To understand the interaction between the polypyrrole matrix and detected analytes, we performed quantum chemical calculations. Here, the polymer is described by two layers separated by an optimized distance (Figure 7). 

A supercell large enough to contain the molecules (3 × 3 × 1) is built and a vacuum size of 9 Å is set along the z axis in order to trap a considered molecule between the PPy layers. This method is quite different from those generally cited in the literature, including ours [21], as PPy was modeled by a layer and not by fragments (monomer, dimers, etc.). The system is fully optimized and Gly molecules were placed, respectively, taking into account the possible interaction sites.

**Figure 8:**
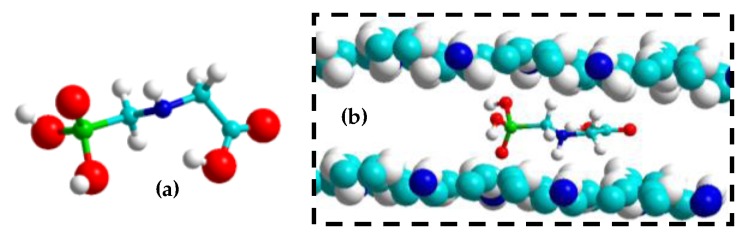
(**a**) Optimized geometries of Gly. (**b**) Most stable configurations of Gly inside the PPy matrix.

The interaction between Gly and polypyrrole is schematized in Figure 8. It appears that Gly can interact with PPy matrix via hydrogen bonds. 

The interaction energy ΔE is calculated from the difference of energies between the complex (E_PPy + Gly_) and those of isolated molecule (E_Gly_) and polymer (E_PPy_), according to Equation (3):

∆E_int_ = E_(PPy + Gly)_ − [E_(PPy)_ + E_(Gly)_] (3)

ΔE was estimated at −145 kJ/mol. This high exothermic value corresponds to two strong hydrogen bonds coupled to the stacking between matrix planes that approach during the complexation.

### 3.5. Selectivity Tests

Selectivity tests were investigated with different pesticides, including gluphosinate ammonium, simetryn, terbuthylazine-2-hydroxy, omethoate, and methidathion. At low concentrations (inferior to 0.1 μM), the responses of each of these pesticides were comparable to those for nonspecific adsorptions observed with NIP. They were, therefore, negligible, within measurement uncertainty. However, we conducted experiments with high concentrations, even if improbable.

**Figure 9:**
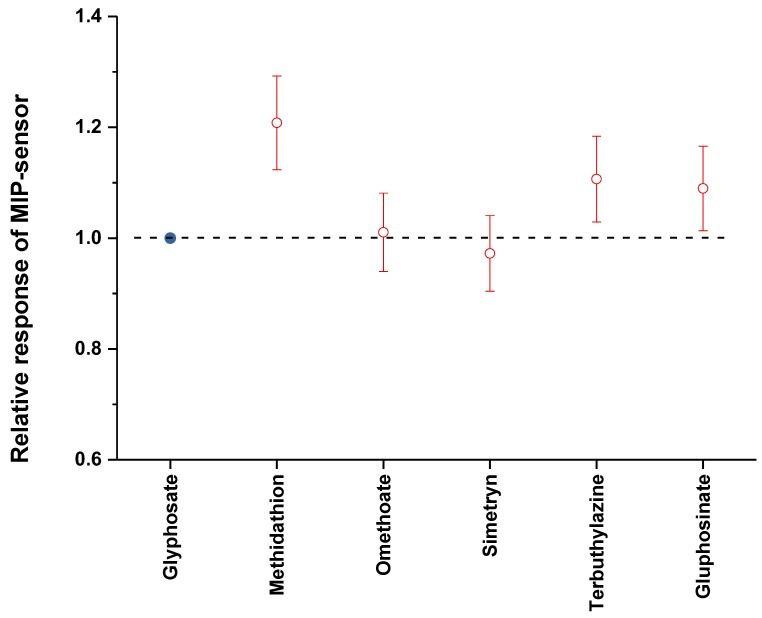
Relative electrochemical responses of MIP-sensor to gluphosinate ammonium, simetryn, terbuthylazine-2-hydroxy, omethoate, and methidathion at concentration of 10^−4^ M, compared to glyphosate’s response at 10^−11^ M.

The electrochemical response of the MIP based sensor towards each of these pesticides used at 10^−4^ M has been compared to that obtained for Gly at 10^−11^ M (Figure 9). 

Results gathered in Figure 9 indicate that the realized sensor is highly selective. Indeed, the response for each of the considered pesticides (at such high concentration) is comparable to the response obtained for Gly at low concentration (within uncertainties), except methidathion, for which the difference is of about 20%.

The same experiments were done with the MIP based surface acoustic wave sensors. Results (not presented here) are comparable to the electrochemical ones: no phase shift was recorded after pesticides injection, except for methidathion where a relative phase shift of about 15% was recorded.

The competitivity tests were conducted by electrochemistry using mixture of Gly at 10^−4^ M and each of the five considered pesticides at the same concentration. The recorded values of current peak have been compared to that obtained for Gly alone. The relative deviations were comprised between 1.7% (for omethoate) and 5% (for gluphosinate ammonium), i.e., close to the measure’s uncertainties.

## 4. Conclusions

Complementary surface acoustic wave (SAW) and electrochemical sensors were functionalized with polypyrrol (PPy)-imprinted polymer for the selective detection of Gly. Their corresponding limits of detection (LOD) were on the order of 10^−12^ M, which are among the lowest values ever reported in the literature. Sensitivities of the designed sensors were found on the order of (75 ± 41) µA/nM and (6.9 ± 2.9) × 10^−20^/nM for electrochemical and gravimetric sensors, respectively.

The relevant dissociation constants of PPy matrix/Gly analytes were estimated from the fitting of both phase and peak current variation versus Gly concentration with two sites binding and Hill–one site binding combined model. Calculations indicate that the combined model is the most appropriate as it takes into account both the mechanical restructuration of the PPy polymer at the nanometer scale, and the chemical complementarity between PPy and Gly. According to this model, the apparent dissociation constants calculated from electrochemical measurements were found equal to K_d1_ = (7.1 ± 3.2) × 10^−13^ M and K_d2_ = (1.6 ± 1.4) × 10^−6^ M, whereas those obtained from gravimetric measurements were on the order of K_d1_ = (2.4 ± 0.9) × 10^−12^ M and K_d2_ = (3.0 ± 0.7) × 10^−7^ M. 

DFT quantum chemical calculations were investigated to estimate the interaction energy between Gly and PPy film. ΔE was found of order of −145 kJ/mol. Dissociation constants and DFT calculations highlighted the strong character of the interaction between Gly and polypyrrole, on one hand, and the crucial role of molecular cavities, which are complementary in terms of shape and functional groups to the investigated template, on the other hand. 

Selectivity and competitivity tests were conducted with the most common and used pesticides: gluphosinate ammonium, simetryn, terbuthylazine-2-hydroxy, omethoate, and methidathion. Gravimetric and electrochemical results indicate that both MIP-based sensors are perfectly able to detect Gly without any ambiguity. 

This study paves the way for the use of MIP based sensors as analytical techniques permitting a routine control in environment monitoring.

## Figures and Tables

**Table 1:** Examples of investigated analytical methods for Gly detection and associated limits of detection (LOD) and the associated linear range.

Sample Matrix	Analytical Technique	LOD	Linear Range	Ref.
Ground water	Solid-phase extraction followed by liquid chromatography coupled to tandem mass spectrometry (SPE-LC-MS/MS)	18.9 pM	0.3–3 nM	[8]
Canal water	Liquid chromatography fluorescence (LC-FLD) + tandem mass spectrometry (MS-MS)	0.6 nM	0.6 nM–0.3 µM	[9]
Tap water	High-performance liquid chromatography (HPLC) and ultraviolet spectroscopy	0.4 µm	29.6 µM–0.6 mM	[10]
Tap water and irrigation water	Electrochemiluminescence	0.2 mM	0.2–16.6 mM	[11]
Pearl River water	Fluorescence	47.3 nM	59.1 nM–47.3 µM	[12]
Water	Optical: prism coupling optical waveguide	1.4 nM	1.4–5.0 nM	[13]
	Laser induced fluorescence (LIF)	0.3 nM	0.1 nM–5.0 µM	[14]
	Enzyme-linked immunosorbent assay (ELISA)	0.6 nM	3.2–4.5 nM	[15]
	Ion chromatography—inductively coupled plasma mass spectrometry	4.1 nM	up to 2.4 µM	[16]
		4.1 nM		
Cereals	Fluorescence	71.0 nM	0.1–14.8 µM	[17]
Serum	LC-MS/MS	0.2 µM		[18]
Urine	HPLC with post-column reaction and FD	5.9 nM	-	[19]
	ELISA	5.3 nM	-	[20]

**Table 2:** Examples of MIP-based sensors for Gly detection.

Main Steps for Gly Detection and/or Sensors Construction	Extraction Technique	Analytical Method	LOD/LOQ	Linear Range	Ref.
- Dissolution of Gly+ C_10_H_19_NO_2_ + C_4_H_8_N_2_S in a porogenic solvent	Stirring in NaH_2_PO_4_ during 30 min at room temperature	HPLC + Fluorescence Detection	LOD: 2.5 nM	1.5 nM to 5.9 µM	[42]
- Addition of C_10_H_14_O_4_ cross-linking monomers + C_2_H_4_O + ‎C_8_H_18_OSi_2_					
- Addition of ‎C_13_H_10_O benzophenone (last)			LOQ: 0.8 nM		
- UV Irradiation for 30 min					
- Dissolution of Gly + chloroforme + methanol	Washing with a 1:9 (*v*/*v*) mixture of acetic acid and methanol	Chemi-luminescence	LOD: 0.27 µM	2.96 µM to 0.2 Mm	[43]
- Further addition of C_3_H_4_O_2_, C_10_H_14_O_4_ (EGDMA) and C_8_H_12_N_4_ (AIBN)					
- Degassing of the mixture with nitrogen for 15 min and then polymerization at 60 °C for 24 h					
- Preparation of Fe_3_O_4_ NPs then Fe_3_O_4_ MIP composite	- Methanol and acetic acid (*v/v*, 9:1) solution in Soxhlet extractor	Cyclic Voltammetry	LOD: 10 µM	-	[44]
- Preparation of solution (a) a dispersion of: ‎C_18_H_29_NaO_3_S and Fe_3_O_4_ NPs into methanol-water + Gly+ C_4_H_6_O_2_ (MAA)					
- Preparation of solution (b) Glutaraldehyde (crosslinker) + NH_4_)_2_S_2_O_8_ (initiator of free radical polymerization) in methanol					
- Injection of (b) into (a) in an atmosphere of nitrogen at 60 °C for 18 h					
- Preparation of (HAuCl_4_)	PBS at pH 7.2 for 30 min	Linear sweep voltammetry (LSV)	LOQ: 5 fM	5 fM to 5 nM	[45]
-fnctionalized AuNPs					
- Dissolution of Gly + PATP-functionalized AuNPs + [Fe(CN)_6_]^3−/4−^ in a solution of PBS with 20% methanol					
- Electropolymerization (20 cycles): scan rate = 100 mV/s, voltage from −0.35 to 0.85 V					
- Application of a fixed potential of 0.80 V during 30 min to stabilize the MIP					
- Dissolution of Gly + C_4_H_5_N in PBS.	A mixture of water + Methanol + dichloro-methane + Ultra-Turrax^®^	Electrochemical Impedance Spectroscopy + Cyclic voltammetry	LOD: 0.5 µM	2.4 to 7.1 µM	[46]
- Electropolymerization (20 cycles): scan rate = 0.05 V s^−1^. Voltage ranged from −0.35 to 0.85 V					
- Synthesis of MAC monomers	Acetonitrile + triethylamine (4/1: *v*/*v*) for 30 min	Differential pulse anodic stripping voltammetry (DPASV)	LOD: 2.1 nM	23.5 nM to 0.1 µM	[47]
- Synthesis of GNPs gold nanoparticles					
- Attachment of GNPs to pencil rod (PGE) surface					
- Formation of MAC–gold nanoparticles complexes					
- Dissolution of C_10_H_8_N_2_ + CuCl_2_ in DMSO to get a solution of Cu(II)-complex					
- Mixture of this complex with NGLY templates + GLU + EGDMA + MWCNTs + (C_2_H_5_)_3_N reducing agent					
- Purge of the whole mixture in a glass tube with N_2_ gas					
- Spin coating at 2500 rpm onto the surface of MAC modified GNPs-PGE					
- Incubation in an oven for 3 h at 70 °C					
**Electro-polymerization of Py + Gly + LiClO_4_ by chronoamperometry**	**Methanol + Acetic acid 1/1: *v*/*v***	**Square wave voltammetry and gravimetry**	**LOD: 1 pM**	**1 pM to 1 nM**	**This study**
